# Three decades of telemedicine in Brazil: Mapping the regulatory framework from 1990 to 2018

**DOI:** 10.1371/journal.pone.0242869

**Published:** 2020-11-25

**Authors:** Angélica Baptista Silva, Rondineli Mendes da Silva, Gizele da Rocha Ribeiro, Ana Cristina Carneiro Menezes Guedes, Daniela Lacerda Santos, Carla Cardi Nepomuceno, Rosângela Caetano

**Affiliations:** 1 Department of Human Rights, Health and Cultural Diversity, Sergio Arouca National School of Public Health, Oswaldo Cruz Foundation, Rio de Janeiro, Rio de Janeiro, Brazil; 2 Department of Medicines and Pharmaceutical Services Policies, Sergio Arouca National School of Public Health, Oswaldo Cruz Foundation, Rio de Janeiro, Rio de Janeiro, Brazil; 3 Institute of Scientific and Technological Communication and Information in Health, Oswaldo Cruz Foundation, Rio de Janeiro, Rio de Janeiro, Brazil; 4 Telehealth Unit, Department of Research and Teaching, Federal Hospital of State Employees, Rio de Janeiro, Rio de Janeiro, Brazil; 5 Department of Collective Health, Medical School of Petrópolis, Faculdade Arthur Sá Earp Neto, Petrópolis, Rio de Janeiro, Brazil; 6 Department of Health Policy, Planning and Administration, Institute of Social Medicine, Universidade do Estado do Rio de Janeiro, Rio de Janeiro, Rio de Janeiro, Brazil; University of Oklahoma Health Sciences Center, UNITED STATES

## Abstract

This study characterized the evolution of Brazilian public telemedicine policy in the Brazilian Unified Health System for 30 years from 1988 to 2019 by analyzing its legal framework. We identified 79 telemedicine-related legislations from the federal government (laws, decrees, and ordinances) and 31 regulations of federal councils of health professionals. Three historical phases were established according to the public policy cycle, and material was classified according to the purpose of the normative documents. The content analysis was based on the advocacy coalition framework model. Of the federal legislations, 8.9% were for the Formulation/Decision-Making phase, 43% for the Organization/Implementation phase, and 48.1% for the Expansion/Maturation phase of telemedicine policy in Brazil. The Federal Council of Medicine was the most active in standardizing telemedicine and was responsible for 21 (67.7%) regulations. The first legislations were passed in 2000; however, the coalitions discussed topics related to telemedicine and created their belief systems from the 1990’s. The time cycle which included formulation and decision making for Brazilian telemedicine policy, extended until 2007 with the creation of several technical working groups. The expansion and maturation of telemedicine services began in 2011 with the decentralization of telemedicine policy actions across the country. Telemedicine centers which performed telediagnosis influenced the computerization of primary health care units. We conclude that Brazilian telemedicine field has greatly grown and changed in recent years. However, despite the proliferation of legislations and regulations in the period studied, there is still no fully consolidated process for setting up a wholly defined regulatory framework for telemedicine in Brazil.

## Introduction

Information and communications technology (ICT) have entirely transformed healthcare by circumventing space and time limitations. Aiming to promote individual and community health, ICT has expanded to progressively include information exchange for the diagnosis, treatment, and prevention of diseases; research and evaluation; and continuing education of health professionals and users [1, 2]. It is increasingly viewed as an important tool for addressing the contemporary challenges of health systems.

Modern society first referred to this set of events as “telemedicine.” Telemedicine generally refers to the use of telecommunications to provide clinical care from a distance, challenging the assumption that care requires physical presence and contact between professionals and patients. Telemedicine is the oldest term in the literature and is indexed in the *Medical Subject Headings* (MeSH). Telehealth derives from telemedicine but has a broader focus on health promotion and education and includes essential care professional areas such as nursing, pharmacy, and rehabilitation. However, the two terms are often used interchangeably [3].

With the emergence and expansion of the internet, other terms such as electronic health (e-health), mobile health (m-health), and ubiquitous health (u-health) emerged [4]. As the internet incorporated a new protocol that communicates with sensors at the beginning of the 21^st^ century, the theme of Internet of Things (IoT) and health emerged.

The World Health Organization (WHO), which created an observatory for electronic health in 2005, has advocated for regulation of these activities in national territories and named this set of activities digital health in 2018 [5]. From the 1950s to 2020, telemedicine travelled a long semantic path before the name digital health was adopted. However, the turning point in its conceptualization was the change from “telemedicine” to “telehealth,” which incorporates the paradigm of health promotion and a more comprehensive health field in ICT.

Positive aspects of telemedicine use in health care services include (i) reduced costs and time, as there is no need to transport patients; (ii) increased efficiency in health resource management due to evaluation and screening by specialists; (iii) faster access to specialists in emergencies; (iv) reduced hospital admissions; (v) more efficient use of resources through the decentralization of assistance, expanding the services to more people; (vi) possibility of increased cooperation among researchers via shared clinical records; and (vii) higher quality of educational programs for doctors and other health professionals located outside specialized centers [2].

Despite these advantages and almost seventy years of history, further dissemination of telemedicine still faces several technical, legal, ethical, regulatory, and cultural challenges across the globe and in Brazil [6–8].

Brazilian telemedicine may be an essential component of medical care when considering some characteristics of Brazil and its health system. Brazil is the fifth largest country in the world, with almost 8.52 million km^2^ of territorial extension and more than 210 million inhabitants as of 2020 [9]. It is a federative republic with three autonomous levels of government: the federal government, 26 states and the Federal District, and 5,570 municipalities, of which 1,451 (26%) have less than 10,000 inhabitants. The country is divided into five geographic macroregions: North, Northeast, Central-West, Southeast, and South. States in the South and Southeast regions are generally more urban and industrialized with better infrastructure compared to those in the North and Northeast regions, which are poorer and lack health resources.

The 1988 Brazilian Constitution defined health as a universal right and responsibility of the state. The public health system (Unified Health System, abbreviated as SUS in Portuguese) is based on three principles: the universal right to comprehensive health care at all levels of complexity (primary, secondary, and tertiary), decentralization with responsibilities given to the three levels of government (federal, state, and municipal), and social participation in formulating and monitoring the implementation of health policies. Tax revenues and social contributions from all three levels of government finance the public health system. Nearly 25% of Brazilians, comprising mostly middle- and higher-income residents, have additional private health insurance coverage or pay directly for services [10]. In 2017, the health expenditure in Brazil was 9.4% of the gross domestic product (GDP), of which public spending accounted for 41.8%; health expenditure per capita corresponded to US$ 928.799 [11]. The country has significant internal health inequities, with a heterogeneous distribution of infrastructure and differences in the qualifications of professionals. There are several rural areas and remote territories with scarce health resources and gaps in medical care. SUS implementation resulted in a significant and rapid increase in primary care services, especially after establishment of the Family Health Program in 1994, as well as organization of health networks involving emergency care and mental health services. The number of family health teams progressively increased, raising the provision of services from 4% to 62% of the population. However, access to specialized care remains a challenge with unmet demands, long wait times, and delays in diagnosis and treatment [12]. In Brazil, there are approximately 2.18 physicians for every thousand inhabitants. This indicator differs significantly between regions, materializing a picture of inequality at the geographic level and between states, capitals, and municipalities in the interior. The distribution of specialists is more uneven than that of physicians [13]. In 2017, Brazil had 7,514 hospital units, but the number of establishments (3.7%) and beds (8.4%) have decreased since 2009. The country has 1.72 hospital beds per thousand inhabitants with significant differences between regions varying from 1.55 in the North to 2.08 in the South [14]. In terms of ICT infrastructure, 90% of health care providers in the public sector used computers, and 77% had access to the internet in 2017. Approximately 19,000 of the 42,600 primary care units used electronic health records [15].

In this context, telemedicine can be a critical element to realize SUS principles, minimize regional inequalities in the distribution of health resources and referring specialists, facilitate second opinions for specialized or rare clinical cases, and establish continuing education for health professionals.

In Brazil, telemedicine emerged in a decentralized and fragmented manner in health, teaching, and research establishments in the 1990s. In 1994, the Ministry of Health together with the Brazilian Association of Public Health (Associação Brasileira de Saúde Coletiva) held a workshop on the use and dissemination of health information to develop information policy for SUS. This final document described the fragmentation of health information systems, the type of standardization of large databases in the area, and the impact of computerization on health care [16]. Working and discussion groups multiplied in parallel with the growth of isolated initiatives in university spaces and health services. Among these groups, a working group instituted by the Ministry of Health in May 2000 with members of several government agencies was formed to study, discuss, and propose a policy related to telemedicine use at the national level [17, 18].

In the second half of the 2000s, two public administration initiatives integrated part of these isolated activities into two central parts of the existing telemedicine structure in Brazil: the Telemedicine University Network (Rede Universitária de Telemedicina), known as RUTE, and the Telehealth Brazil Networks Program (Programa Telessaúde Brasil Redes).

RUTE was created in 2006 by the Brazilian Ministry of Science and Technology to deploy communication infrastructure at public universities, university hospitals, health institutions, and certified teaching and research hospitals [19]. RUTE connects the services developed in university hospitals to professionals in distant cities through the sharing of medical records, consultations, exams, and second opinions. As of June 2019, RUTE has 139 units in operation in all macroregions of Brazil, covering over 150 institutions and 55 Special Interest Groups (SIGs). SIGs are the main activity of RUTE and exemplify the integration between telemedicine initiatives that it promotes. In these groups, health professionals discuss specific topics on videoconferences focused on teaching and research or provide care at a distance (when a second medical opinion is required) [20].

In 2007, the Ministry of Health created the Telehealth Brazil Networks Program as a pilot project (initially called the Brazilian Telehealth Program) in nine states across all macroregions of the country (Ordinance no. 35/2007) [21]. Initially, this involved nine telehealth centers located in public universities connected to 900 basic health units in 728 municipalities, which were located mainly in remote and isolated areas. This project offered teleconsulting, telediagnosis, and tele-education in primary care services [22]. With Ordinance no. 402 of 2010, the pilot project was formally recognized as a program and was physically expanded to other Brazilian states while maintaining primary health care (PHC) connections [23]. In October 2011, Ordinance no. 2546 expanded this initiative to more complex services as a tool to manage and strengthen health care networks. Its name changed to the Telehealth Brazil Networks Program (Programa Nacional Telessaúde Brasil Redes), and it expanded to include telemedicine in healthcare procedure coding list of SUS [24].

In addition to these two initiatives, the Open University of SUS (Universidade Aberta do SUS—UNA-SUS) was instituted by Decree no. 7385/2010 and offered training and continuing education to SUS health professionals in the distance modality [25]. The UNA-SUS has more than 2 million enrollments in the 36 public higher education institutions that make up the system. Today, educational offerings cover approximately 98% of Brazilian municipalities, with approximately 50% of trained professionals in PHC [26].

Moreover, telemedicine of health services linked to private health insurance and plans also expanded. Its entry into this health subsector in the first decade of the 21st century broadened its reach to the entire Brazilian health system [27]. It should be noted that remote medical and other professional health consultations via ICTs were only allowed in Brazil after the spread of the coronavirus disease (COVID-19), even for exceptional cases (Law no. 13.989, April 2020) [28]. There was already a demand for medical teleconsultations, which were seen as a novel and promising segment of the health services market in Brazil [29].

A legal framework is necessary to acquire infrastructure, authorize and integrate into the health care system, and reimburse telemedicine for providing patient care. Thus, it is necessary to investigate other factors beyond technological infrastructure that facilitate reduced distances and waiting time. A central question is how telemedicine is established in different societies and the laws by which it is regulated. Thus, we aim to understand how telemedicine was established in legal terms in Brazil over the last 30 years.

Discussions on social and ethical issues such as licensing, privacy, security, confidentiality, and scope of practice have not kept up with the discovery of new devices, applications, and services involving telemedicine. There are few studies on laws and specific regulations for implementing telemedicine [30]. Multisector public policies concerning protection of data and confidentiality have been outlined in countries and economic blocs, such as the European Union’s Data Protection Directive [31]. However, it is still necessary to identify and examine the set of rules for telemedicine policy in SUS management. In Brazil, there are councils recognized and authorized by the State which regulate the professional activities of each specific health category. The Federal Council of Medicine (FCM), for instance, has regulated doctors since 1957 [32]. Furthermore, since telemedicine is an area that involves multidisciplinary activities and requires interdisciplinary action, several professional councils have discussed and established regulations for telemedicine in Brazil.

This study characterizes the evolution of Brazilian telemedicine policy in the public health system by analyzing governmental legal documents and incorporating regulations of the health professionals’ councils.

## Materials and methods

This is an exploratory, retrospective, descriptive study on the legal framework of telemedicine in the SUS. Two sets of documents were examined: the federal government legislation related to telemedicine (laws, decrees, and ordinances) and the regulations (in the form of resolutions and technical notes) of professional councils.

The study spanned from January 1990 to December 2018 because the law institutionalizing SUS is from September 1990 (Law no 8080) [33], and the first telemedicine-related initiatives in Brazil emerged in that decade.

The primary data sources used were legislations from the Brazilian Ministry of Health (Ministério da Saúde—MoH) and Ministry of Science, Technology, Innovations, and Communications (Ministério da Ciência, Tecnologia, Inovações e Comunicações—MSTIC). These two ministries conceived and led the organization of Brazilian telemedicine policies and practice.

For this purpose, a search was carried out in the Health Legislation System Saúde Legis with the support of a librarian trained in information science (GRR). The Saúde Legis contains all legal documents related to SUS at the Brazilian federal level and is available at: http://portal2.saude.gov.br/saudelegis/leg_norma_pesq_consulta.cfm?limpar=pesquisa. We used the keywords “*Telemedicina*, *Telesaúde*, *Telessaúde*, *e-Saúde*” in the “subject” field of Saúde Legis without limiting the type of publication and start date with results through December 2018. The search was conducted on 7 and 8 January 2019. Similarly, a search for legislations was carried out in the MSTIC portal, (http://www.mctic.gov.br/mctic/opencms/legislacao/index.html) using the same keywords as those used in Saúde Legis. In several instances, other relevant legislations were mentioned in the body of laws, decrees, ordinances, and resolutions. In such case, all additional citations of the legislation were verified for inclusion and minimization of eventual losses.

Public documents were searched for and retrieved from the MoH, RUTE and telehealth centers electronic portals to improve the sensitivity of searching for information and filling gaps due to generic keywords.

The inclusion criterion was a legal document published in 1990 or later. All documents identified in the legislation searches were retrieved and read in full-text to verify their focus on telehealth, telemedicine, or electronic health and related to issues of legislation, regulation, financing, organization and standardization of activities, modalities, services, and policies related to the field of study.

**Fig 1:**
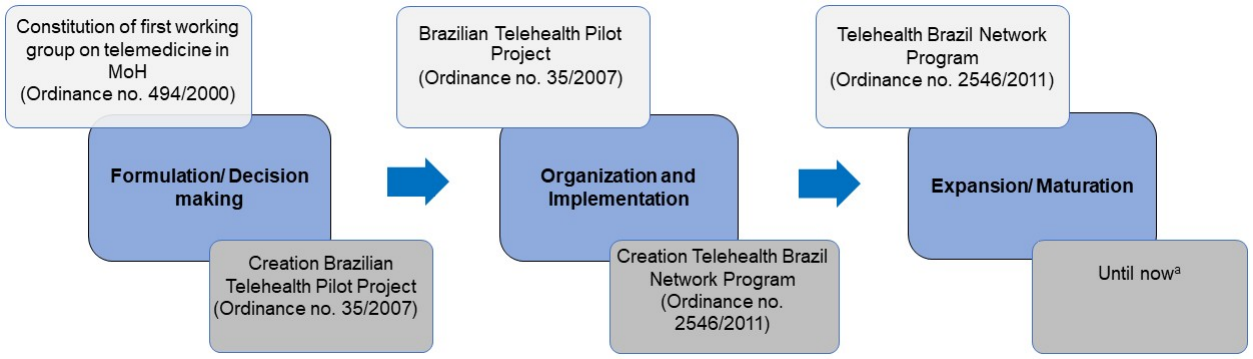
Historical phases of telemedicine development in Brazil. MoH—Ministry of Health. ^a^ Although the Expansion/Maturation phase extends to the present day, data collection was completed in December 2018.

Documents identified in the federal legislation systems were categorized according to the historical phases of telemedicine development in Brazil. Three phases were pre-established based on the historical evolution and the decision-making process for telemedicine [34, 35]. The public policy cycle was considered, models were adapted from those previously used by Khoja et al. (2013) [36] in e-health and Haddad et al. (2016) [37] in development of the Telehealth Brazil Networks Program. The three phases with their historical landmarks are (1) Formulation/Decision Making, which corresponds to telemedicine precursors and fragmented initiatives in Brazil; (2) Organization/Implementation, which comprises creation of geographically restricted telehealth pilot projects until constitution of the Telehealth Brazil Networks Program; and (3) Expansion/Maturation, which covers expansion of this program to more Brazilian states and municipalities (Fig 1).

The purpose of each legislation was classified into one of four nonexclusive and pre-established categories. The categories were adapted from the classification proposed by Silva et al. (2019) [38] in their investigation of cancer care policy in Brazil. Table 1 describes the classification categories. Depending on their content, legislations could be classified in more than one category.

**Table 1:** Categories used in classifying the purposes of telemedicine-related federal legislations and regulations in Brazil.

Purpose	Description
Structuring Rules	It involved the organization of the health system and services; management/governance; functional parameters; physical facilities; criteria for accreditation of services; forms of qualification; human resources; evaluation parameters; and support systems.
Enablement/Accreditation	It included the insertion of establishments qualified to conduct telemedicine.
Funding	It encompassed the cost and programming of resources. It also included transfer models, payments, and procedural amounts.
Protocols and Technology	It included operational definitions and standardizations; data interoperability; and privacy and confidentiality policy.

Adapted from Silva et al. [38].

Classification was carried out by two independent researchers (RMS and ABS) trained to minimize subjectivity in establishing the historical phase and purpose of the material. Disagreements in classifications were discussed and resolved through consensus. The Kappa quotient was used to quantify the degree of agreement in the classifications of the two researchers.

The health sector graduate professional categories in Brazil were identified from a search in the Brazilian Classification of Occupations (Classificação Brasileira de Ocupações—CBO) [39] and the National Health Council [40]. The CBO is the standard for the recognition and codification of titles and contents of occupations in the Brazilian labor market. The National Health Council (Conselho Nacional de Saúde), an integral part of MoH's organizational structure, recognizes graduate health professions in Brazil because of one of its legal statutory attributions. Fourteen health professions and their respective federal professional councils at the federal regulation level were identified. A manual search was conducted on the websites of the following professional councils to identify regulations related to telemedicine: Medicine, Nursing, Psychology, Pharmacy, Dentistry, Physical Education, Veterinary Medicine, Physiotherapy, Nutrition, Speech Therapy, Social Work, Biology, Biomedicine, and Occupational Therapy. Professional councils at the state level were not searched, as regulation at the national level was our primary interest. All records through 31 December 2018 were included. The keywords were the same as those used when searching for legislation in Saúde Legis. The regulations identified on professional councils’ websites were also categorized according to purpose with the classifications shown in Table 1.

A descriptive analysis of the publication year, historical phase, and purpose of the legislation/regulation was conducted. The evolution of historical phases was characterized within each purpose category. Stata^®^ software (Version 12, StataCorp) was used to assess the correlation between historical phase and legislation purpose. Fisher’s exact test with a significance level of 5% was used.

The advocacy coalition framework (ACF) [41, 42] assumes that understanding policy change processes and the associated role of policy learning requires observation of at least a decade, that the appropriate unit of analysis for the study of this change is through political subsystems, that these subsystems include intergovernmental dimensions, that public policies or programs can be conceptualized by belief systems (sets of priorities and consensus on how to achieve them), and a leading role of scientific/technical information in policy change. This model helped the comprehension of the legislations and regulations telemedicine-related implemented during SUS. In addition to the normative aspect, the legislations and regulations establishing rights and duties cover a set of beliefs and values of interest groups comprising the coalitions. In this context, legislations are documents that formalize the conflicting ideas.

The analysis of legal framework according to chronology, origin, object, and purposes aimed to clarify the implementation and consolidation of telemedicine public policy in Brazil. The analysis helped to identify political subsystems contributing to policy over 30 years and characterize the maturity of telemedicine services as a government program.

## Results

**Fig 2:**
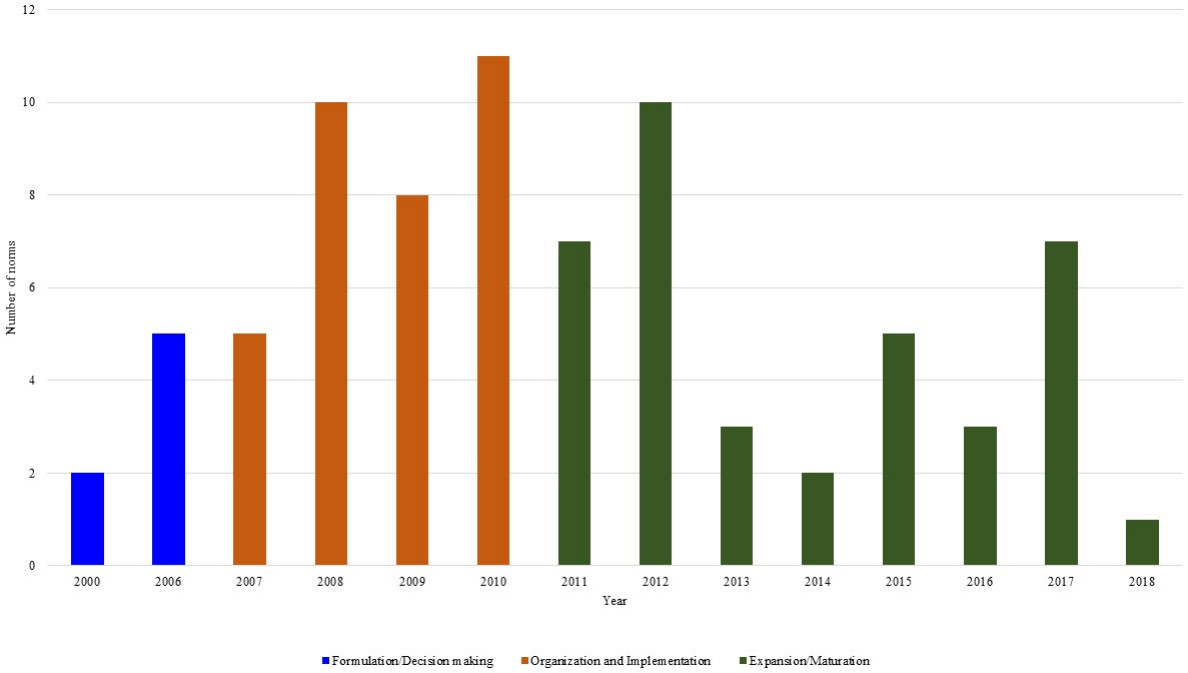
Telemedicine-related federal legislation according to historical phases in Brazil, 1990-2018^a^. ^a^ The period studied spanned from 1990 to 2018, but no legislation was found until 2000.

No federal legislations addressing telemedicine in the 1990s were identified. We identified 79 telemedicine-related legislations in the period studied, with 77 from the MoH and two from the MSTIC. The first legislations were introduced in 2000 with the publication of Ordinances no. 494 and no. 531, and both organize working groups on telemedicine in the MoH [17, 18]. Between 2000 and 2005, no further legislations were published (Fig 2). Additionally, 31 regulations were identified from the federal Councils of health professionals and are presented separately below.

Only seven federal legislations (8.9%) were related to the initial phase of Formulation/Decision Making. The Organization/Implementation stage accounted for 43% of the legislations. The Expansion/Maturation phase of telemedicine policy in Brazil accounted for approximately half (48.1%) of the 79 legal documents (Fig 2).

Formulation/Decision making began in the 2000s and was characterized by the official creation of working groups to study, discuss, and prepare proposals for the adoption of telemedicine in Brazil. In 2006, this process advanced within the MoH which created the Permanent Telehealth Commission to advise the Ministry on project development and preparation of cooperation proposals for financing services, among other activities related to telemedicine [43].

The Organization/Implementation phase started with a pilot project that created telehealth centers in federal and state public universities in nine Brazilian states (Amazonas, Ceará, Pernambuco, Goiás, Minas Gerais, Rio de Janeiro, São Paulo, Santa Catarina, and Rio Grande do Sul) distributed in the five geographic regions (Ordinance no. 35 of 2007) [21]. Thus, the link between telemedicine actions and PHC was established. This link was subsequently reinforced with Ordinance no. 402/2010 [23], which extended the initiative to the national level. The last two years studied preceding the Telehealth Brazil Networks Program accounted for 56% of the legislations in this phase.

The Expansion/Maturation phase began with implementation of the Telehealth Brazil Networks Program in October 2011 [24]. The next year had the most legislations (10/38, 26.3%), reflecting the need for legal order for financing the program and its rules in Brazil (Fig 2).

**Fig 3:**
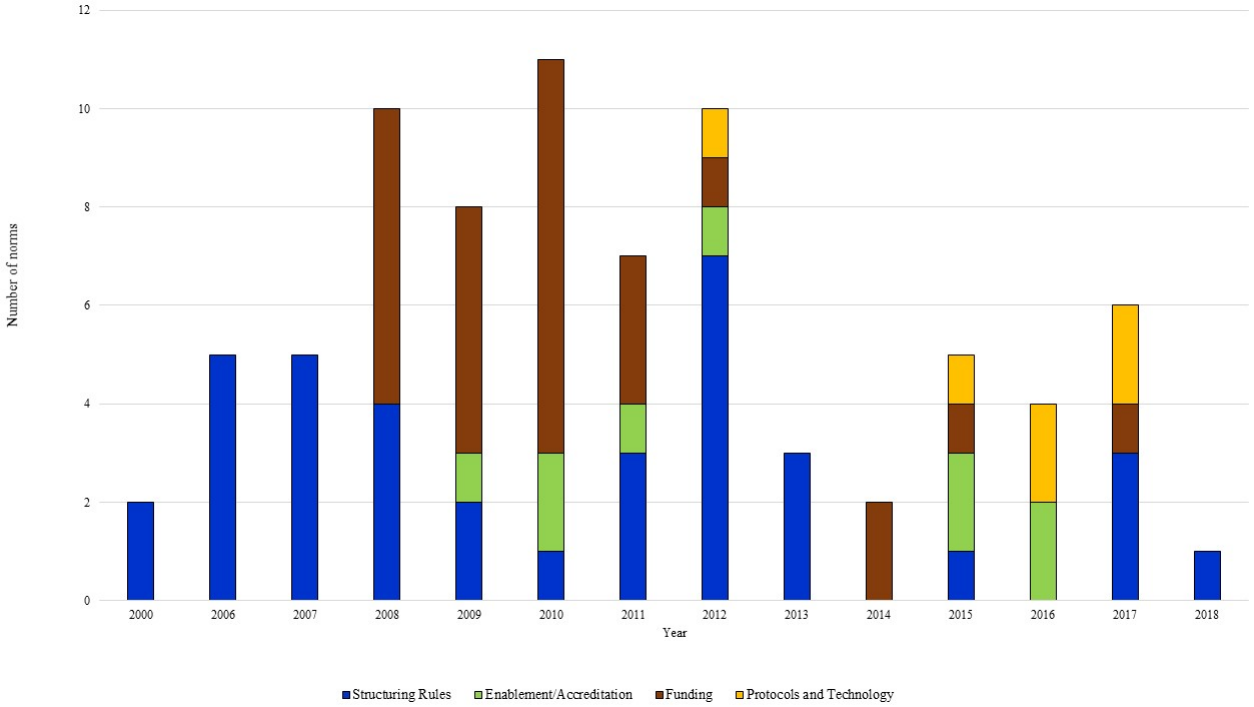
Telemedicine-related federal legislation according purpose, 2000- 2018^a^. ^a^ The period studied corresponds to 1990 to 2018, but no legislation was found until 2000.

Regarding the purpose of norms identified, 37 (46.8%) federal legislations were related to structuring rules, 27 (34.2%) to funding, 9 (11.4%) to habilitation/accreditation, and 6 (7.6%) to protocols and technology. The reliability analysis for classification of legislation purpose showed good agreement between the two researchers (Kappa = 0.66). There was an association between the phase and purpose of federal legislation (p = 0.01). Legislations related to structuring rules for telemedicine was present in all years except 2014 and 2016; 18 (48.6%) of them occurred in the Expansion/Maturation phase of telemedicine policy. The Organization/Implementation phase contained the most (19; 70.4%) regulations related to financing activities and services. Legislations regarding operational definitions, system standardization, data interoperability, and confidentiality and privacy policies were present only in the most advanced stage of telemedicine policy development (Fig 3).

Examination of the 31 regulations from the health professional councils highlighted the FCM as the most active in standardizing aspects related to telemedicine in Brazil, as it was responsible for 21 (67.7%) of the regulations. The vast majority (95.2%) of FCM regulations were related to structural aspects. Only four other professional councils had telemedicine regulations before 2018 (Table 2).

**Table 2:** Telemedicine-related regulations of federal professional councils according to their purpose, 1990 to 2018.

Federal Professional Councils	No. Regulations	Purpose	No. Purposes
Nursing	1	Structuring Rules	1
Speech Therapy	2	Structuring Rules	2
Medicine	21	Protocols and Technology	1
		Structuring Rules	20
Dentistry^a^	5	Structuring Rules	5
		Funding	2
Psychology	2	Structuring Rules	2
**Total**	**31**	**―**	**33**

Note:

^a^ Five regulations were identified for the Federal Council of Dentistry. However, two regulations had two purposes (structuring rules and financing).

The first FCM regulation related to telemedicine was passed in 1998 and referred to urgent and emergency medical activities in their prehospital phase [44]. This is also regulated under the latest version of the Medical Code of Ethics. The Code refers to specific applications (teleradiology, telecardiology, and teledermatology) and other elements related to electronic health records [45]. The Federal Councils of Psychology, Dentistry, and Speech Therapy also regulated the provision of services through ICT with various forms of informational and communicative methods via the internet, telephone devices, combined or hybrid devices, websites, applications, digital platforms, etc. The Federal Nursing Council prohibits nurses from complying with remote medical prescriptions provided by radio, telephones, SMS messages, electronic mail, social media networks, or other means for which the doctor’s stamp and signature are not available. However, the Council makes an exception to this rule for telemedicine medical prescription [46].

**Fig 4:**
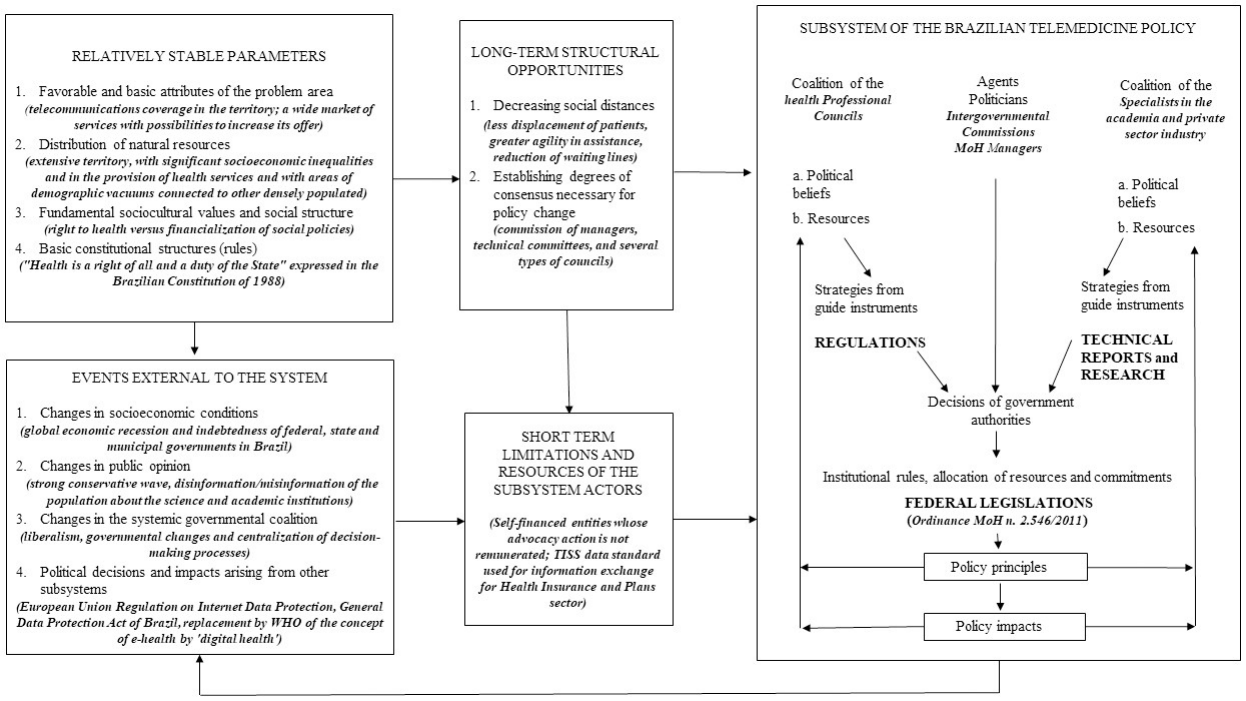
Diagram of advocacy coalitions for telemedicine policy in the Brazilian Health Sector, 1990–2018. WHO, World Health Organization; TISS–*Troca de Informações na Saúde Suplementar* is the Exchange of Information in the Health Insurance and Plans; MoH, Ministry of Health.

The Advocacy Coalition Framework (ACF) proposed by Sabatier and Jenkins-Smith [41, 42] explains the emergence of and changes in public policies due to belief systems and disputes between different actors of a policy subsystem. The results of attempts to apply the model to the developing telemedicine subsystem in Brazil over 30 years are illustrated in Fig 4.

The ACF considers the decisions underlying policies to be partially determined by a set of parameters that are relatively stable over time, over which coalitions have little control. The constitutional principle of “*Health is a right of all citizens and a duty of the State*” defined a political framework for the need for health actions and services. The extension of Brazilian territory and the existing assistance gaps posed challenges for the health services supply and favored the emergence and expansion of telemedicine in Brazil. A telecommunications coverage network in part of the Brazilian territory facilitated this dissemination (Fig 4).

Among the structural opportunities that contributed to telemedicine development was its potential to promote less geographical displacement of patients and reducing waiting times especially for medical specialties. Regarding the degree of consensus required for establishment of telemedicine policy in Brazil, the creation of several commissions and working groups with broad participation by different policy actors from the Formulation/Decision-making phase stands out. These actors included ministries such as MoH, MSTIC, and the Ministry of Education, international organizations such as the Pan American Health Organization, and several institutions such as the Federal Council of Medicine and federal universities (Fig 4).

The coalition diagram shows a top-down policy flow in which the Brazilian federal government is the principal political agent establishing institutional rules, allocation of financial and human resources, and commitments of states and municipalities of the telemedicine subsystem. Ordinance no. 2546/2011 [24], later replaced by Consolidation Ordinance no. 5/2017 [47], created the Telehealth Brazil Networks Program. It promoted alignment of telemedicine activities with the strategic objectives of MoH and established the basis of telemedicine services (Fig 4).

## Discussion

Analysis of the legal framework for a specific policy helps clarify how that policy emerged and evolved. It provides a basis for understanding how telemedicine was integrated into the Brazilian political agenda and how some proposals and initiatives were successfully implemented, providing insights into how interactions between context, institutions, policy actors, and interest groups shaped the formulation and implementation of this policy.

The study did not identify any federal legislation in the 1990s, and the first were ordinances that created working groups in telemedicine in MoH in 2000 [17, 18]. However, the lack of legislation does not mean that isolated initiatives did not exist in the 1990s. During this period, the first coalitions of policy actors and interest groups began to form mainly in universities and academic environments. At the turn of the decade, the National Education and Research Network (RNP) was created by the Ministry of Science and Technology to build a national academic internet infrastructure. In 1991, RNP implemented the first backbone for internet in Brazil. Later, in 2006, the RNP invited 19 institutions to create a health project involving telemedicine, which came to be known as the Telemedicine University Network (RUTE) that implemented interconnection infrastructure in university hospitals [19]. Other initiatives included telemedicine discussions between the National Health Council (a collegiate body that aimed at engaging civil society in the decision making and formulation of health policies) with the newly created Brazilian Telecommunications Agency, which was responsible for rules for establishing and sharing telecommunication infrastructure [48]. Another example is the creation of telediagnostic projects in public universities [49].

The organization/implementation and expansion/maturation phases accounted for the vast majority (91%) of telemedicine-related federal legislations in Brazil. During the implementation phase, there was a progressive geographical and scope expansion of the newly created Brazilian Telehealth Program. The transition from development to implementation stages involved many national-level institutions, as well as subnational commissions at different levels. The legislation of this period organized telemedicine structure at the federal, state, and municipal levels, ensuring governance of the program [37]. The Expansion/Maturation phase legislations promote the operation of telemedicine services, regulate the transfer of financial resources, and guarantee the provision of human resources for program execution in the states and municipalities. These government measures determined telemedicine policy progress in Brazil.

The objectives of the federal legislations were statistically correlated with the historical phases. Legislations covering the entire spectrum of purposes were passed in the expansion/maturation phase. This phase also accounted for approximately half (47.4%) of the legislation designed to structure Brazilian telemedicine policy.

Some examples of structural federal legislation are worth mentioning. A typology for telemedicine services was established in Ordinance no. 2546/2011, the same ordinance that created the Telehealth Brazil Networks Program [24]. An establishment is classified as telemedicine if ICT is used to provide health care and education across geographical and temporal distances, and it may be public or private. The services and the description of their modalities (telediagnosis, teleconsultation, etc.) need to be registered as a health establishment in the National Registry System of Health Establishments (Sistema de Cadastro Nacional de Estabelecimentos de Saúde—SCNES). Each type of service has a specific code, a condition for its habilitation, and subsequent payment for the actions performed, which were covered in several regulations in the Expansion/Maturation phase [50].

Several studies described and evaluated the activities of telemedicine services mostly in university hospitals in the capitals [51, 52]. Telemedicine centers provided service to primary care teams, comprising tele-education, telediagnosis, telehealthcare, and formative second opinion. Initiatives at the state and municipal levels were created for local management. Entry into SCNES denotes the need for funds and sustainability of the service, with a list of auditory health procedures, diagnosis by graphical methods, ophthalmology, among others, directly involving a range of specialized professionals such as dermatologists, radiologists, speech therapists, pediatricians, otolaryngologists, neurologists, pulmonologists, and their respective support teams [53].

The MoH and the Ministry of Education included telemedicine services in postgraduate medical residency and multiprofessional residency programs [54, 55]. Telemedicine was also integrated into national continuing education programs for primary care health professionals related to treatment of chronic diseases, prevention and treatment of obesity, and smoking cessation [56, 57].

In addition to Primary Care, the Urgency and Emergency Care Network also incorporated telemedicine during this period. Telemedicine became part of the teleregulation processes for urgent and emergency care and prehospital medical mobile care in municipalities and regions [58]. Additionally, telemedicine was integrated into specific lines of medical care. For example, the stroke line regulated neurological care at a distance in 2012 [59], aiming to cover neurological care within 30 minutes of patient’s admission [60]. A similar movement was observed in other urgent and emergency areas, such as care for acute myocardial infarction [61, 62] and pediatric cardiology [63].

Among the various specialized services provided by telemedicine, telemammography stands out as a telediagnostic service established in a federal ordinance in 2010 [64]. Telemammography helped to overcome gaps in the distribution of mammography in Brazil, impacting breast cancer screening and women’s health care [65].

Subsystems encompass individual and institutional agents in coalitions that make policies. In addition to the federal public administration, professional councils are another essential component of the Brazilian telemedicine policy subsystem. The professional categories of medicine and dentistry established the most telemedicine regulations in the period studied, placing professionals in their respective functions. It should be noted that doctors and dentists held management positions during structuring and expansion of the National Telehealth Brazil Networks Program [66], contributed relevant scientific knowledge on the subject, and helped consolidate telemedicine symbolically and culturally [51, 66]. Nursing has also played a leading role in the introduction of information technologies in healthcare [67]. Although responsible for several studies on this subject in Brazil [68–70], the Nursing Federal Council generated few regulations detected in this analysis, which requires further investigation.

FCM, responsible for 63.6% of the professional regulations identified here, judges and disciplines the medical profession through supervisory and regulatory action and regulates doctors’ participation telemedicine-related activities throughout Brazil. For example, FCM defined that the services offered by telemedicine need to be registered in the Council with a list of participating doctors and that the FCM should establish surveillance and evaluation of techniques concerning the quality of care, doctor-patient relationship, and preservation of professional confidentiality [45].

This Council is an excellent example of conflicting positions within the same policy actor weighing on coalitions of interest. For example, the revocation of Resolution FCM no. 2227 of 6 February 2019 defined telemedicine as a way to provide ICT-mediated medical services, authorizing medical teleconsultations in the country and regulating surgery robotics, among other subjects [71, 72]. The revocation was due to mobilization of part of the medical category and to declare resistance of some state professional councils, which claimed that there was little discussion among professionals and that financial interests overshadowed medical ethics [73]. With the FCM’s decision to repeal Resolution 2227, medical consultation using telemedicine remained not permitted in Brazil.

This situation only recently changed due to the coronavirus pandemic, under considerable reluctance from the FCM. Based on Law no. 13979/2020 [74], MoH published Ordinance no. 467 on 23 March 2020, providing operationalization of measures to deal with the epidemic, which allows teleconsultation, in an exceptional character, in the SUS health services and private sector [75]. Finally, on 15 April 2020, Law no. 13,989 was sanctioned, authorizing the use of telemedicine in any health activities in Brazil, including teleconsultation, while the COVID-19 epidemic lasts [76].

In 2003, a study carried out by the National Regulatory Agency for Health Insurance and Plans (Agência Nacional de Saúde Suplementar—ANS) created a mandatory standard for the Exchange of Information in the Health Insurance and Plans (Troca de Informações na Saúde Suplementar—TISS). TISS subsidized the political beliefs and resources of several specialists in the ICT industry to regulate telemedicine and electronic registration in establishments [77]. In 2012, this Agency regulated the TISS, whose aim was to encourage the adoption of national information standards, unique terminology, and unambiguous identifiers to allow interoperability between different public information systems and health systems [78]. Its creators had the political conviction to adopt it as a national standard related to the electronic exchange of information, whose original proposal already provided unequivocal identification of users (called National Health Card System—Sistema de Cartão Nacional de Saúde) and health establishments (SCNES) [79].

There was a collaboration in which different advocacy coalitions were formed in the telemedicine policy subsystem in the SUS. The organization and implementation of telemedicine policy in Brazil took place mainly between 2008 and 2010, with diversified funding through public funds. National economic evaluation studies, such as those of cost-effectiveness, report that a high initial investment is required for implementation of telemedicine services, and it is often difficult to convince management to fund the first instance [80]. Although some claim that this cost is diluted over years [81], there is no convincing evidence to conclude whether the use of telemedicine technologies demonstrate value at an acceptable level of investment [82].

2011 was an important moment in the telemedicine expansion/maturation phase in Brazil. For example, the government invested in educating the workforce involved in telemedicine services during this time [54]. A systematic review on strengthening health services using ICT found it strategic to invest in human resources to implement and support telemedicine [83].

An external event corresponding to the political subsystem at the international level was assembly of the World Health Organization, which approved a resolution that conceptualizes digital health as an activity for Sustainable Development Goals in the member states, support of national health systems in promoting health and preventing diseases, and improvement in accessibility and quality of health services [5].

Several documents demonstrated the political beliefs and resources of coalition experts. These documents include the Health Information and Information Technology Directive Plans (Planos Diretores de Informação e Tecnologia da Informação em Saúde) [84], the Telehealth Manual for Primary Care (Manual de Telessaúde para Atenção Básica) [85], and the Methodological Guide for Telehealth Programs and Services (Guia Metodológico para Programas e Serviços em Telessaúde) [86]. All of these documents have the common objective of promoting guidelines for implementation of telemedicine services and establishment of a legal framework for telemedicine.

The resolution approved by the WHO and the documents influenced the current rules, especially the National Health Information and Informatics Policy [87] and its developments such as the e-Health strategy for Brazil [88], which was approved by the Tripartite Intergovernmental Commission. This commission brings together the health secretaries of municipalities, states, and the MoH, with significant influence of the coalition of experts from the ICT industry and academia.

Another recent legislation outside of the period studied (May 2019) [89] assigns to the Department of Regulation, Evaluation, and Control, which is linked to the Department of Specialized Care, the responsibility of managing the content and structure of information models, business rules and administrative and clinical terminologies of health care related to actions, health services and health establishments, and care and correlates; definition, management, and maintenance of the health terminologies repositories; and corroboration of internal departmental dispute (Health Monitoring and Assessment/DEMAS, Digital Health/DSD and Informatic Departments of SUS/DATASUS) for the MoH. This context is increasing in the COVID-19 pandemic which requires rapid decisions and massive use of video consultations [90].

Our study examined federal government legislations and professional council regulations over 30 years. One study limitation was restricting the analysis of documents without using qualitative techniques to scrutinize the characteristics and movements of coalitions and policy actors in the design and construction of the telemedicine policy in Brazil. More in-depth studies with these techniques should be conducted in the future to explore the beliefs and interests of coalitions that formed around Brazilian telemedicine. Additionally, research did not include legislations from Brazilian states and municipalities, which may also legislate on telemedicine in their territorial areas.

## Conclusion

Analysis of the legal framework for a specific policy helps clarify how that policy emerged and evolved. This analysis provided a basis for understanding how telemedicine was integrated into the Brazilian political agenda and how some proposals and initiatives were successfully implemented, providing insights into how interactions between context, institutions, policy actors, and interest groups shaped the formulation and implementation of this policy in Brazil.

Since 2011, Brazil has experienced an expansion and maturation of the policy for implementation of information and communication technologies in the Brazilian Unified Health System. However, the Brazilian telemedicine policy is still in the process of improvement, since the set of priorities and consensus on how to carry out the related activities is still the subject of discussion and dispute by several interest groups, internal and external to the MoH.

Despite the proliferation of laws and regulations in the period studied, the process of establishing a legal framework for telemedicine in Brazil does not appear to have been properly concluded. The publication of laws related to teleconsultation during the Coronavirus pandemic in 2020 justifies this statement. Another example is the Brazilian Public Health Information and Informatics Policy, which is still under discussion and approval. These examples indicate that the field is still in configuration, which is expected to remain quite intense in the coming years.

This dynamic points to the relevance of futures studies examining the impacts of legislation still being drafted as well as the impacts on the development of models that assess the maturity of Brazilian telemedicine services and their integration into the health system.

## Supporting information

S1 Table Characteristics of total federal legislations and regulations from professional councils. (XLSX)
